# Dermoscopy and Onychomycosis: guided nail abrasion for mycological samples*

**DOI:** 10.1590/abd1806-4841.20154615

**Published:** 2015

**Authors:** Diego Leonardo Bet, Ana Lucia dos Reis, Nilton Di Chiacchio, Walter Belda Junior

**Affiliations:** 1 Hospital do Servidor Público Municipal de São Paulo (HSPM-SP) - São Paulo (SP), Brazil; 2 Universidade de São Paulo (USP) - São Paulo (SP), Brazil

**Keywords:** Dermoscopy, Mycology, Nail diseases, Onychomycosis

## Abstract

Mycological examination is still the cornerstone for the diagnosis of onychomycosis for many dermatologists, but sampling technique interferes on its sensitivity and specificity. Nail abrasion may be used to reach the most proximal part of the lesion and can be easily accomplished with an electric abrasor. We suggest nail plate dermoscopy to identify the best location for localized abrasion to obtain adequate samples for mycological examination.

Nail fungal infections are considered the most common in onychopathy in adults.^1^ It may represent up to 50% of all nail lesions and laboratory confirmation of the fungal etiology is required for the diagnosis. Mycological examination with KOH preparation and fungal cultures are commonly used for this purpose^2^. Their sensitivity and specificity are respectively between 72-80% / 72-76% for KOH and 20-53% / 82-100% for fungal cultures, that may vary significantly when performed by an experienced mycologist with proper sampling technique.^3,4^ Nail scrapings obtained at the distal part of the nail are often positive for fungi and bacterial contaminants present in the "gateway" of the nail lesion left behind by the real pathogen. Therefore, it is known that sampling for mycological examination should be performed at the most proximal portion of the nail lesion, where there is a higher probability to find the fungus responsible for the nail invasion.^5^ However it may be uncomfortable and even painful for the patient, because it requires progression of instruments under the nail plate. Localized abrasion is a technique that allows obtaining suitable material of the proximal part of the lesion through a vertical piercing on the nail plate, with little or no discomfort for the patient.^6^

Dermoscopy patterns for onychomycosis were described by Piraccini et al ^7^, showing high sensitivity and specificity to differentiate onychomycosis from traumatic onycholysis. Onychomycosis cases demonstrated jagged edges with longitudinal streaks, while lesions diagnosed as traumatic onycholysis had linear edges without spikes.

We demonstrate the utility of dermoscopy to guide local abrasion to obtain better quality samples in a case of a 58 year-old female patient with two previous negative mycological tests, performed by an experienced mycologist, on a toenail lesion (Figure 1). On dermatoscopic examination we found an irregular longitudinal white streaked pattern with a yellowish central spike, suggestive of onychomycosis (Figure 2).^7^ We conducted a vertical perforation with an electric drill at this central spike until the local resistance decreased and the nail became brittle. Direct examination with KOH of this sample showed hyaline septate hyphae (Figures 3 and 4), and *Trichophyton rubrum* grew in culture confirming the diagnosis of onychomycosis.

**Figure 1:**
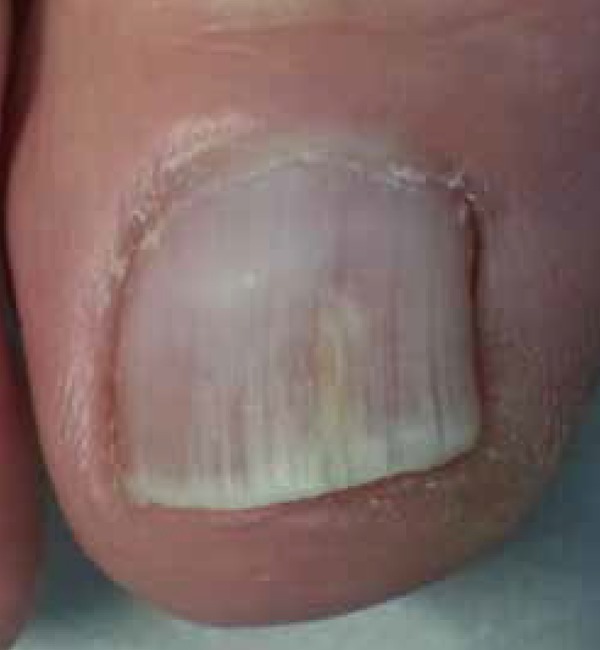
Clinically suspected toenail onychomycosis with negative mycological results.

**Figure 2:**
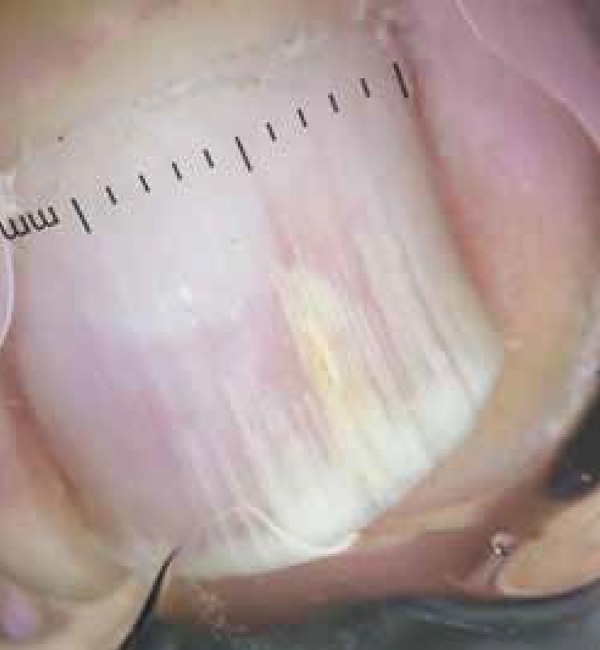
Dermoscopy of the nail plate showing longitudinal irregular white streaks. Near the center a thick yellowish streak is seen.

**Figure 3:**
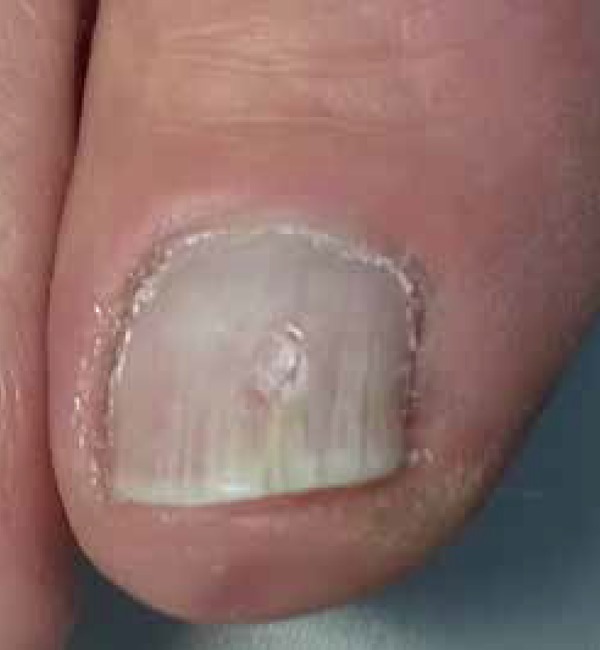
Nail abrasion guided by dermoscopy performed at the tip of the yellow streak.

**Figure 4:**
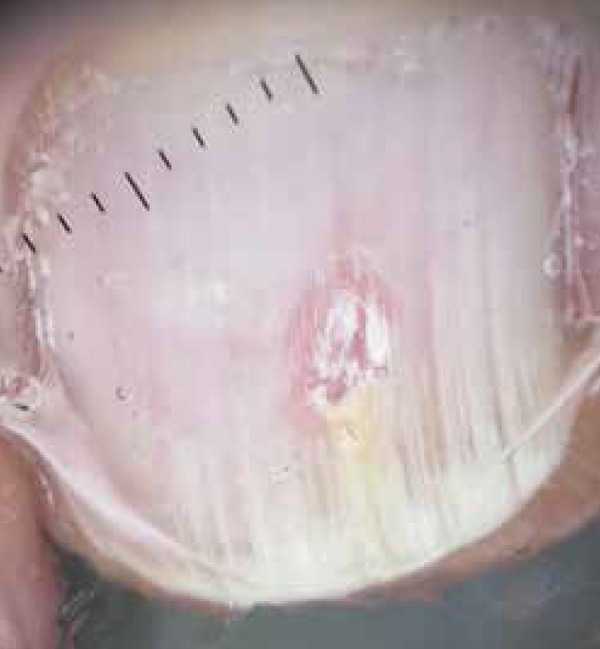
Nail plate dermoscopy showing the vertical pierce after abrasion. Mycological samples from this area were positive.

Mycological examination still remains the cornerstone for onychomycosis diagnosis and portable electric abrasors are suitable for use in in-office sampling. Dermoscopy is already part of the dermatologist diagnostic routine and, in our experience, an useful tool to locate the best proximal site for mycological sampling thru abrasion.
